# S50. EMPLOYING TEXT-MESSAGES TO IMPROVE MOTIVATION: MOBILE ENHANCEMENT OF MOTIVATION IN SCHIZOPHRENIA

**DOI:** 10.1093/schbul/sby018.837

**Published:** 2018-04-01

**Authors:** Lauren Luther, Melanie Watkins, Annalee Johnson-Kwochka, Michelle Salyers

**Affiliations:** Indiana University-Purdue University

## Abstract

**Background:**

Motivation deficits are among the strongest determinants of reduced functioning and quality of life in people with schizophrenia. Mobile interventions are a promising approach to improving these deficits because they can provide frequent cues and reinforcements to support goal-directed behavior in daily life. The objective of this study is to assess the initial feasibility/acceptability and effectiveness of Mobile Enhancement of Motivation in Schizophrenia (MEMS), a personalized mobile text message intervention, compared to a goal-setting alone intervention.

**Methods:**

Fifty-six participants with a schizophrenia-spectrum disorder have been enrolled in this ongoing controlled pilot study. Twenty-seven participants have been randomized to MEMS, while 29 participants have been randomized to the goal-setting alone condition. Participants in both groups set individualized recovery goals to complete over an 8-week period. Those in the MEMS group also receive three sets of personalized, interactive text messages each weekday to reinforce and cue goal completion. Blinded assessments are conducted before and after the 8-week period and include validated measures of motivation, quality of life, and functioning. Goal attainment and self-reported satisfaction with MEMS are also assessed.

**Results:**

To date, 36 participants (n = 18 in each group) have completed both baseline and follow-up assessments. Initial results suggest that relative to the goal-setting alone group, the MEMS group demonstrated significantly greater improvements in clinician-rated motivation (F(1, 33) = 7.14, p = .01; between-group d = .89). Specifically, the MEMS group demonstrated significantly higher clinician-rated motivation after 8 weeks (within-group d = .62), while clinician-rated motivation remained the same in the goal-setting alone group (within-group d = -.02). Across both groups, participants also significantly improved on clinician-rated functioning over time (t(35) = -2.56, p = .02, d = .43), but there was no difference between the two groups (F(1, 33) = .01, p = .94; between-group d = .03). No improvement on self-reported quality of life was observed in either group or across the full sample. The MEMS group reported strong satisfaction with the text-messages. Recruitment has been completed, and analyses from the full sample will be ready to present at the meeting.

**Discussion:**

Initial results indicate that MEMS is acceptable and may successfully improve motivation in people with schizophrenia-spectrum disorders. However, additional analyses with the full sample are needed to more rigorously test the feasibility and effectiveness of MEMS.

